# Especially Concerning the Country and Small Town General Practitioner

**Published:** 1915-01

**Authors:** C. H. Hughes

**Affiliations:** St. Louis, Mo.


### EDITORIAL DEPARTMENT.

MRS. F. E. DANIEL, Publisher and Managing Editor.

DR. R. H. L. BIBB, Associate Editor.

CONTRIBUTING EDITORS:

Dr. H. Sheridan Baketel, New York, editor *Medical Times:* Dr. M. H. Boerner, Austin, Texas: Dr. G. Henri Bogart, Paris, Ill.; Dr. M. M. Carrick, Dallas, Texas; Dr. Edward H. Carey, Dean Medical Department, Baylor University, Dallas, Texas; Dr. W. L. Crosthwaite, Waco, Texas; Dr. T. D. Crothers, Hartford, Conn.: Dr. H. W. Cummings, Hearne, Texas; Dr. Oscar Dowling, New Orleans, President Louisiana State Board of Health; Havelock Ellis, M. D., London, Eng.; Dr. May Agnes Hopkins, Dallas, Texas; Dr A. W. Fly, Galveston, Texas; Dr. G. B. Foscue, Waco, Texas; Dr. E. S. Goodhue, Holualoa, Kona, Hawaii; Dr. M. B. Grace, Seguin, Texas; Dr. Joe Gilbert. Austin, Texas; Dr. Charles W. Hughes, St. Louis, editor *Alienist and Neurologist*: Dr. James G. Kiernan, Chicago; Dr. George H. Lee, Galveston, Texas; Dr. John T. Moore, Houston, Texas; Dr. Frank Paschal, San Antonio, Texas; Dr. John Preston, Austin, Texas, Superintendent State Lunatic Asylum; Dr. G. Wilse Robinson, Kansas City, Mo.; Dr. Bacon Saunders, Fort Worth, Texas; Dr. Allen J. Smith, Philadelphia, Medical Department, University of Pennsylvania; Dr. Z. T. Scott, Austin, Texas; Dr. Ralph Steiner, Austin, Texas, President Texas State Board of Health; Dr. Walter S. Sutton, Kansas City, Mo.; Dr. John S. Turner, Dallas, Texas; Dr. Charles Venable, San Antonio, Texas.

---

### Especially Concerning the Country and Small Town General Practitioner.

The country doctor is the most reliant of physicians, and must be the broadest gauged in his practice. He surveys the entire field of diagnosis and resource. In his student days and after study later years he considers diagnostic and therapeutic utility value in what he acquires of knowledge. He searches for the practical in biology, chemistry, pathology and therapy. He gathers knowledge from every source and stores it in his mind, condensed for use in relief or cure and understanding of disease.

A doctor once, a long way from a large city where chemists were plentiful, was suddenly called upon to treat a patient for acute results of lead poisoning at the house he was visiting. In his saddle-bags was a bottle of aromatic sulphuric acid, used to make a bisulphate soluble mixture of quinia, which he always carried and often administered, for the country was very malarious. He knew something about chemical reagents, incompatibles and toxic salts rendered inert by combination with right chemical agents. He made a sulphuric acid lemonade and gave it to the poisoned patient until the sugar of lead was rendered inert, insoluble and harmless by the resulting sulphate of lead which resulted from the combination, and the patient was cured.

The country doctor going to the post-graduate school may be likened to the country merchant going to the great city to select his goods for the needs of his patrons at home. Likewise the medical student, in the old days, when he spent two years making the professional rounds with his preceptor. Both had and have an idea of what they should bring back with them, and they should both be so taught now.

It is obvious that no doctor can select and carry all the knowledge of the biological and chemical and microscopical laboratories (nor of the specialties), for the field of every department of research tributary to medicine (general and special) is too extensive. The country doctor must be intelligently selective in accumulating curative and ready diagnostic sources for the medical welfare of his community in chemistry, hematology, hemo-therapy, serum therapy, galenical, alkaloidal, etc.—Written for the "Red Back" by C. H. Hughes, M: D., St. Louis, Mo.

